# (*E*)-Ethyl *N*′-(4-bromo­benzyl­idene)hydrazinecarboxyl­ate

**DOI:** 10.1107/S1600536808023763

**Published:** 2008-07-31

**Authors:** Bo Gao

**Affiliations:** a Marine College, Zhejiang Institute of Communications, Hangzhou 311112, People’s Republic of China

## Abstract

The title compound, C_10_H_11_BrN_2_O_2_, crystallizes with two independent mol­ecules in the asymmetric unit, in which the dihedral angles between the benzene ring and the hydrazine carboxylic acid mean plane are 3.0 (4) and 45.3 (3)°. The mol­ecules are linked into a one-dimensional network by inter­molecular N—H⋯O hydrogen bonds.

## Related literature

For general background, see: Parashar *et al.* (1988 ▶); Hadjoudis *et al.*(1987 ▶); Borg *et al.* (1999 ▶). For a related structure, see: Shang *et al.* (2007 ▶).
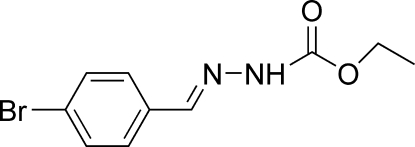


## Experimental

### 

#### Crystal data

C_10_H_11_BrN_2_O_2_
*M* *_r_* = 271.11
Monoclinic, 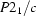

*a* = 16.499 (3) Å
*b* = 8.6052 (19) Å
*c* = 18.277 (4) Å
β = 116.279 (7)°
*V* = 2326.7 (8) Å^3^
*Z* = 8
Mo *K*α radiation
μ = 3.52 mm^−1^
*T* = 123 (2) K
0.30 × 0.26 × 0.25 mm

#### Data collection

Bruker SMART CCD diffractometer
Absorption correction: multi-scan (*SADABS*; Bruker, 2002 ▶) *T*_min_ = 0.419, *T*_max_ = 0.474 (expected range = 0.367–0.415)
24092 measured reflections
4075 independent reflections
1989 reflections with *I* > 2σ(*I*)
*R*_int_ = 0.139

#### Refinement

*R*[*F* ^2^ > 2σ(*F* ^2^)] = 0.093
*wR*(*F* ^2^) = 0.259
*S* = 0.88
4075 reflections
273 parameters
H-atom parameters constrained
Δρ_max_ = 1.35 e Å^−3^
Δρ_min_ = −1.10 e Å^−3^

### 

Data collection: *SMART* (Bruker, 2002 ▶); cell refinement: *SAINT* (Bruker, 2002 ▶); data reduction: *SAINT*; program(s) used to solve structure: *SHELXS97* (Sheldrick, 2008 ▶); program(s) used to refine structure: *SHELXL97* (Sheldrick, 2008 ▶); molecular graphics: *SHELXTL* (Sheldrick, 2008 ▶); software used to prepare material for publication: *SHELXTL*.

## Supplementary Material

Crystal structure: contains datablocks I, global. DOI: 10.1107/S1600536808023763/hb2762sup1.cif

Structure factors: contains datablocks I. DOI: 10.1107/S1600536808023763/hb2762Isup2.hkl

Additional supplementary materials:  crystallographic information; 3D view; checkCIF report

## Figures and Tables

**Table 1:** Hydrogen-bond geometry (Å, °)

*D*—H⋯*A*	*D*—H	H⋯*A*	*D*⋯*A*	*D*—H⋯*A*
N2—H2*A*⋯O3	0.86	2.09	2.913 (7)	161
N4—H4*A*⋯O1^i^	0.86	2.09	2.875 (7)	152

Symmetry code: (i) 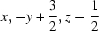.

**Fig. 1.**
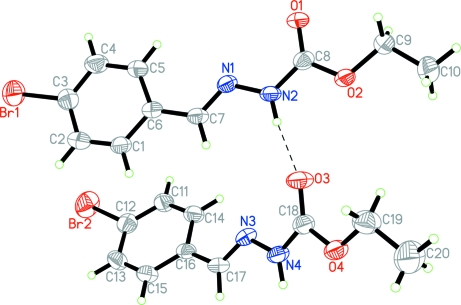
Molecular structure of (I), showing 30% probability displacement ellipsoids for the non-hydrogen atoms. The hydrogen bond is shown as a dashed line.

**Fig. 2.**
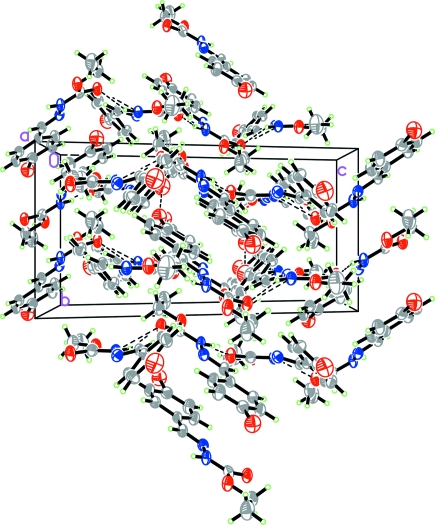
The crystal packing of (I). Hydrogen bonds are shown as dashed lines.

## supplementary crystallographic information

### Comment

Benzaldehydehydrazone derivatives have received considerable attention for a long time due to their pharmacological activity (Parashar *et al.*, 1988) and their photochromic properties (Hadjoudis *et al.*, 1987). They are important intermidiates for 1,3,4-oxadiazoles, which have been reported to be versatile compounds with many properties (Borg *et al.*, 1999). As a further investigation of this type of derivatives, the crystal structure of the title compound, (I), is reported here (Fig. 1).

Compound (I) crystallizes with two independent but essentially identical molecules in the asymmetric unit. Each independent molecule adopts a *trans* configuration with respect to the C═N bond. In each molecule, the hydrazine carboxylic acid methyl ester group is twisted away from the attached ring. The dihedral angle between C1-C6 and N1/N2/O1/O2/C8-C10 planes is 3.0 (4)°  and that between C11-C16 and N3/N4/O3/O4/C18 planes is 45.3 (3)°. The bond lengths and angles of each molecule in the asymmetric unit agree with those observed for methyl N'-[(E)-4-methoxybenzylidene]hydrazinecarboxylate (Shang *et al.*, 2007).

In the crystal of (I), the molecules are linked into a one-dimensional network by intermolecular N—H···O hydrogen bonds (Table 1, Fig. 2).

### Experimental

4-Bromobenzaldehyde (1.84 g, 0.01 mol) and ethyl hydrazinecarboxylate (1.04g, 0.01 mol) were dissolved in stirred methanol (20 ml) and left for 3 h at room temperature. The resulting solid was filtered off and recrystallized from ethanol to give the title compound in 85% yield. Colourless blocks of (I) were obtained by slow evaporation of a methanol solution at room temperature (m.p. 433–435 K).

### Refinement

The H atoms were positioned geometrically (N-H = 0.86 Å and C-H = 0.95-0.99Å) and refined as riding, with *U*_iso_(H) = 1.2–1.5*U*_eq_(carrier).

### Crystal data

**Table:**

C_10_H_11_BrN_2_O_2_	*F*_000_ = 1088
*M**_r_* = 271.11	*D*_x_ = 1.548 Mg m^−^^3^
Monoclinic, *P*2_1_/*c*	Mo *K*α radiation λ = 0.71073 Å
Hall symbol: -P 2ybc	Cell parameters from 4075 reflections
*a* = 16.499 (3) Å	θ = 1.4–25.0º
*b* = 8.6052 (19) Å	µ = 3.52 mm^−^^1^
*c* = 18.277 (4) Å	*T* = 123 (2) K
β = 116.279 (7)º	Block, colourless
*V* = 2326.7 (8) Å^3^	0.30 × 0.26 × 0.25 mm
*Z* = 8	

### Data collection

**Table:**

Bruker SMART CCD diffractometer	4075 independent reflections
Radiation source: fine-focus sealed tube	1989 reflections with *I* > 2σ(*I*)
Monochromator: graphite	*R*_int_ = 0.139
*T* = 123(2) K	θ_max_ = 25.0º
ω scans	θ_min_ = 1.4º
Absorption correction: multi-scan(SADABS; Bruker, 2002)	*h* = −19→18
*T*_min_ = 0.419, *T*_max_ = 0.474	*k* = −9→10
24092 measured reflections	*l* = −21→21

### Refinement

**Table:**

Refinement on *F*^2^	Secondary atom site location: difference Fourier map
Least-squares matrix: full	Hydrogen site location: inferred from neighbouring sites
*R*[*F*^2^ > 2σ(*F*^2^)] = 0.093	H-atom parameters constrained
*wR*(*F*^2^) = 0.259	*w* = 1/[σ^2^(*F*_o_^2^) + (0.1543*P*)^2^] where *P* = (*F*_o_^2^ + 2*F*_c_^2^)/3
*S* = 0.88	(Δ/σ)_max_ < 0.001
4075 reflections	Δρ_max_ = 1.35 e Å^−^^3^
273 parameters	Δρ_min_ = −1.10 e Å^−^^3^
Primary atom site location: structure-invariant direct methods	Extinction correction: none

### Special details

**Table:**

Geometry. All esds (except the esd in the dihedral angle between two l.s. planes) are estimated using the full covariance matrix. The cell esds are taken into account individually in the estimation of esds in distances, angles and torsion angles; correlations between esds in cell parameters are only used when they are defined by crystal symmetry. An approximate (isotropic) treatment of cell esds is used for estimating esds involving l.s. planes.
Refinement. Refinement of F^2^ against ALL reflections. The weighted R-factor wR and goodness of fit S are based on F^2^, conventional R-factors R are based on F, with F set to zero for negative F^2^. The threshold expression of F^2^ > 2sigma(F^2^) is used only for calculating R-factors(gt) etc. and is not relevant to the choice of reflections for refinement. R-factors based on F^2^ are statistically about twice as large as those based on F, and R- factors based on ALL data will be even larger.

### Fractional atomic coordinates and isotropic or equivalent isotropic displacement parameters (Å^2^)

**Table:**

	*x*	*y*	*z*	*U*_iso_*/*U*_eq_	
Br1	−0.38953 (6)	0.41924 (14)	0.35560 (6)	0.1017 (5)	
O1	0.1467 (3)	0.9272 (6)	0.6334 (3)	0.0683 (15)	
O2	0.2401 (3)	0.9406 (6)	0.5725 (3)	0.0683 (14)	
N1	0.0294 (4)	0.7619 (7)	0.4990 (3)	0.0547 (14)	
N2	0.1102 (4)	0.8180 (7)	0.5084 (3)	0.0591 (16)	
H2A	0.1271	0.8016	0.4708	0.071*	
C1	−0.1496 (5)	0.5277 (9)	0.3484 (4)	0.0614 (19)	
H1	−0.1226	0.5078	0.3130	0.074*	
C2	−0.2340 (6)	0.4681 (9)	0.3288 (4)	0.068 (2)	
H2	−0.2657	0.4103	0.2801	0.082*	
C3	−0.2716 (5)	0.4947 (10)	0.3822 (5)	0.069 (2)	
C4	−0.2254 (6)	0.5766 (10)	0.4538 (5)	0.079 (2)	
H4	−0.2513	0.5928	0.4905	0.094*	
C5	−0.1413 (5)	0.6341 (10)	0.4709 (4)	0.069 (2)	
H5	−0.1087	0.6882	0.5208	0.082*	
C6	−0.1020 (5)	0.6165 (8)	0.4185 (4)	0.0540 (17)	
C7	−0.0147 (5)	0.6797 (9)	0.4356 (4)	0.0566 (18)	
H7	0.0106	0.6598	0.3988	0.068*	
C8	0.1647 (5)	0.8967 (9)	0.5765 (4)	0.0593 (19)	
C9	0.3059 (5)	1.0219 (11)	0.6442 (4)	0.075 (2)	
H9A	0.3253	0.9547	0.6930	0.090*	
H9B	0.2789	1.1178	0.6539	0.090*	
C10	0.3843 (6)	1.0610 (15)	0.6288 (6)	0.113 (4)	
H10A	0.4283	1.1208	0.6749	0.170*	
H10B	0.3639	1.1230	0.5788	0.170*	
H10C	0.4126	0.9650	0.6225	0.170*	
Br2	−0.40506 (7)	0.72172 (16)	0.13867 (7)	0.1115 (5)	
O3	0.2037 (4)	0.7286 (7)	0.4120 (3)	0.0764 (16)	
O4	0.2853 (3)	0.6993 (6)	0.3405 (3)	0.0672 (14)	
N3	0.0511 (4)	0.7304 (7)	0.2633 (3)	0.0555 (15)	
N4	0.1369 (4)	0.7146 (7)	0.2717 (3)	0.0589 (15)	
H4A	0.1461	0.7048	0.2291	0.071*	
C11	−0.2224 (5)	0.8208 (9)	0.2063 (5)	0.065 (2)	
H11	−0.2410	0.8950	0.2342	0.078*	
C12	−0.2835 (6)	0.7232 (10)	0.1526 (5)	0.072 (2)	
C13	−0.2584 (6)	0.6157 (10)	0.1077 (4)	0.074 (2)	
H13	−0.3017	0.5477	0.0693	0.089*	
C14	−0.1333 (5)	0.8142 (9)	0.2212 (4)	0.063 (2)	
H14	−0.0904	0.8821	0.2601	0.075*	
C15	−0.1705 (5)	0.6125 (9)	0.1210 (4)	0.064 (2)	
H15	−0.1532	0.5435	0.0898	0.076*	
C16	−0.1055 (5)	0.7070 (8)	0.1786 (4)	0.0530 (17)	
C17	−0.0116 (5)	0.6937 (8)	0.1946 (4)	0.0549 (18)	
H17	0.0029	0.6569	0.1529	0.066*	
C18	0.2083 (5)	0.7143 (9)	0.3472 (4)	0.0604 (19)	
C19	0.3667 (6)	0.6862 (12)	0.4155 (5)	0.087 (3)	
H19A	0.3691	0.5839	0.4411	0.105*	
H19B	0.3689	0.7686	0.4541	0.105*	
C20	0.4441 (7)	0.7027 (18)	0.3950 (8)	0.134 (5)	
H20A	0.5009	0.6958	0.4451	0.201*	
H20B	0.4406	0.8038	0.3691	0.201*	
H20C	0.4418	0.6196	0.3575	0.201*	

### Atomic displacement parameters (Å^2^)

**Table:**

	*U*^11^	*U*^22^	*U*^33^	*U*^12^	*U*^13^	*U*^23^
Br1	0.0722 (7)	0.1265 (10)	0.1016 (8)	−0.0210 (5)	0.0342 (6)	−0.0047 (6)
O1	0.076 (4)	0.094 (4)	0.044 (3)	−0.011 (3)	0.035 (3)	−0.005 (2)
O2	0.054 (3)	0.110 (4)	0.048 (3)	−0.014 (3)	0.029 (2)	−0.005 (3)
N1	0.053 (4)	0.068 (4)	0.049 (3)	0.001 (3)	0.028 (3)	0.004 (3)
N2	0.053 (4)	0.091 (5)	0.037 (3)	−0.003 (3)	0.024 (3)	−0.005 (3)
C1	0.078 (6)	0.063 (5)	0.051 (4)	−0.001 (4)	0.036 (4)	−0.001 (4)
C2	0.081 (6)	0.073 (5)	0.050 (4)	0.008 (5)	0.028 (4)	−0.003 (4)
C3	0.065 (5)	0.069 (5)	0.065 (5)	0.003 (4)	0.022 (4)	0.005 (4)
C4	0.076 (6)	0.104 (7)	0.068 (5)	0.001 (5)	0.043 (4)	−0.009 (5)
C5	0.072 (5)	0.088 (6)	0.052 (4)	−0.012 (5)	0.033 (4)	−0.011 (4)
C6	0.062 (5)	0.058 (4)	0.044 (4)	0.007 (4)	0.026 (3)	0.004 (3)
C7	0.057 (4)	0.073 (5)	0.044 (4)	0.006 (4)	0.027 (4)	−0.003 (4)
C8	0.052 (4)	0.076 (5)	0.048 (4)	0.005 (4)	0.020 (4)	0.015 (4)
C9	0.063 (5)	0.102 (6)	0.056 (5)	−0.016 (5)	0.023 (4)	0.001 (4)
C10	0.073 (7)	0.173 (12)	0.080 (6)	−0.040 (7)	0.021 (5)	−0.003 (7)
Br2	0.0600 (7)	0.1507 (11)	0.1204 (9)	−0.0002 (6)	0.0369 (6)	0.0102 (7)
O3	0.082 (4)	0.108 (4)	0.050 (3)	0.009 (3)	0.038 (3)	−0.003 (3)
O4	0.054 (3)	0.101 (4)	0.052 (3)	0.004 (3)	0.028 (2)	0.001 (3)
N3	0.058 (4)	0.067 (4)	0.055 (4)	0.005 (3)	0.037 (3)	0.007 (3)
N4	0.062 (4)	0.077 (4)	0.046 (3)	0.001 (3)	0.032 (3)	0.003 (3)
C11	0.063 (5)	0.069 (5)	0.072 (5)	0.002 (4)	0.037 (4)	−0.012 (4)
C12	0.065 (5)	0.085 (6)	0.056 (4)	−0.001 (4)	0.020 (4)	0.014 (4)
C13	0.083 (6)	0.077 (6)	0.050 (4)	−0.015 (5)	0.019 (4)	−0.008 (4)
C14	0.073 (5)	0.066 (5)	0.054 (4)	0.000 (4)	0.032 (4)	−0.007 (4)
C15	0.066 (5)	0.076 (5)	0.049 (4)	0.004 (4)	0.026 (4)	−0.008 (4)
C16	0.069 (5)	0.053 (4)	0.041 (3)	0.003 (4)	0.027 (3)	0.005 (3)
C17	0.065 (5)	0.064 (5)	0.041 (4)	0.009 (4)	0.029 (4)	−0.001 (3)
C18	0.067 (5)	0.064 (5)	0.057 (5)	−0.001 (4)	0.033 (4)	0.002 (4)
C19	0.079 (6)	0.116 (8)	0.063 (5)	0.008 (5)	0.027 (5)	−0.001 (5)
C20	0.065 (7)	0.215 (15)	0.112 (8)	−0.001 (7)	0.030 (6)	−0.005 (9)

### Geometric parameters (Å, °)

**Table:**

Br1—C3	1.899 (8)	Br2—C12	1.907 (8)
O1—C8	1.229 (8)	O3—C18	1.226 (8)
O2—C8	1.333 (9)	O4—C18	1.335 (9)
O2—C9	1.458 (9)	O4—C19	1.437 (10)
N1—C7	1.275 (8)	N3—C17	1.264 (8)
N1—N2	1.355 (8)	N3—N4	1.360 (8)
N2—C8	1.352 (9)	N4—C18	1.361 (9)
N2—H2A	0.8600	N4—H4A	0.8601
C1—C2	1.374 (10)	C11—C12	1.344 (11)
C1—C6	1.396 (10)	C11—C14	1.372 (10)
C1—H1	0.9500	C11—H11	0.9500
C2—C3	1.387 (10)	C12—C13	1.415 (12)
C2—H2	0.9500	C13—C15	1.360 (11)
C3—C4	1.381 (11)	C13—H13	0.9500
C4—C5	1.373 (11)	C14—C16	1.407 (10)
C4—H4	0.9500	C14—H14	0.9500
C5—C6	1.382 (10)	C15—C16	1.386 (10)
C5—H5	0.9500	C15—H15	0.9500
C6—C7	1.439 (10)	C16—C17	1.447 (10)
C7—H7	0.9500	C17—H17	0.9500
C9—C10	1.478 (11)	C19—C20	1.487 (14)
C9—H9A	0.9900	C19—H19A	0.9900
C9—H9B	0.9900	C19—H19B	0.9900
C10—H10A	0.9800	C20—H20A	0.9800
C10—H10B	0.9800	C20—H20B	0.9800
C10—H10C	0.9800	C20—H20C	0.9800
			
C8—O2—C9	115.4 (5)	C18—O4—C19	116.6 (5)
C7—N1—N2	116.5 (5)	C17—N3—N4	116.1 (5)
C8—N2—N1	120.8 (5)	N3—N4—C18	120.3 (5)
C8—N2—H2A	119.9	N3—N4—H4A	120.0
N1—N2—H2A	119.2	C18—N4—H4A	119.7
C2—C1—C6	122.4 (7)	C12—C11—C14	120.7 (7)
C2—C1—H1	118.8	C12—C11—H11	119.6
C6—C1—H1	118.8	C14—C11—H11	119.6
C1—C2—C3	118.2 (7)	C11—C12—C13	120.9 (8)
C1—C2—H2	120.9	C11—C12—Br2	120.4 (7)
C3—C2—H2	120.9	C13—C12—Br2	118.6 (7)
C4—C3—C2	121.3 (8)	C15—C13—C12	118.2 (7)
C4—C3—Br1	119.4 (6)	C15—C13—H13	120.9
C2—C3—Br1	119.4 (6)	C12—C13—H13	120.9
C5—C4—C3	118.6 (7)	C11—C14—C16	120.0 (7)
C5—C4—H4	120.7	C11—C14—H14	120.0
C3—C4—H4	120.7	C16—C14—H14	120.0
C4—C5—C6	122.6 (7)	C13—C15—C16	121.9 (7)
C4—C5—H5	118.7	C13—C15—H15	119.1
C6—C5—H5	118.7	C16—C15—H15	119.1
C5—C6—C1	116.8 (7)	C15—C16—C14	118.2 (7)
C5—C6—C7	122.7 (7)	C15—C16—C17	120.3 (6)
C1—C6—C7	120.5 (6)	C14—C16—C17	121.4 (7)
N1—C7—C6	121.3 (6)	N3—C17—C16	120.9 (6)
N1—C7—H7	119.3	N3—C17—H17	119.6
C6—C7—H7	119.3	C16—C17—H17	119.6
O1—C8—O2	124.6 (7)	O3—C18—O4	124.5 (7)
O1—C8—N2	125.0 (7)	O3—C18—N4	125.6 (7)
O2—C8—N2	110.4 (6)	O4—C18—N4	109.9 (6)
O2—C9—C10	107.7 (7)	O4—C19—C20	107.3 (7)
O2—C9—H9A	110.2	O4—C19—H19A	110.2
C10—C9—H9A	110.2	C20—C19—H19A	110.2
O2—C9—H9B	110.2	O4—C19—H19B	110.2
C10—C9—H9B	110.2	C20—C19—H19B	110.2
H9A—C9—H9B	108.5	H19A—C19—H19B	108.5
C9—C10—H10A	109.5	C19—C20—H20A	109.5
C9—C10—H10B	109.5	C19—C20—H20B	109.5
H10A—C10—H10B	109.5	H20A—C20—H20B	109.5
C9—C10—H10C	109.5	C19—C20—H20C	109.5
H10A—C10—H10C	109.5	H20A—C20—H20C	109.5
H10B—C10—H10C	109.5	H20B—C20—H20C	109.5
			
C7—N1—N2—C8	−175.2 (7)	C17—N3—N4—C18	−164.0 (7)
C6—C1—C2—C3	−1.6 (11)	C14—C11—C12—C13	−2.6 (12)
C1—C2—C3—C4	−1.0 (12)	C14—C11—C12—Br2	175.0 (6)
C1—C2—C3—Br1	178.2 (6)	C11—C12—C13—C15	0.7 (12)
C2—C3—C4—C5	1.0 (12)	Br2—C12—C13—C15	−176.9 (6)
Br1—C3—C4—C5	−178.2 (6)	C12—C11—C14—C16	1.5 (11)
C3—C4—C5—C6	1.6 (13)	C12—C13—C15—C16	2.2 (11)
C4—C5—C6—C1	−4.0 (12)	C13—C15—C16—C14	−3.3 (11)
C4—C5—C6—C7	178.6 (7)	C13—C15—C16—C17	176.2 (7)
C2—C1—C6—C5	4.0 (11)	C11—C14—C16—C15	1.4 (10)
C2—C1—C6—C7	−178.5 (7)	C11—C14—C16—C17	−178.1 (7)
N2—N1—C7—C6	−178.9 (6)	N4—N3—C17—C16	−179.5 (6)
C5—C6—C7—N1	−3.2 (11)	C15—C16—C17—N3	−153.1 (7)
C1—C6—C7—N1	179.5 (7)	C14—C16—C17—N3	26.4 (10)
C9—O2—C8—O1	3.9 (10)	C19—O4—C18—O3	5.5 (11)
C9—O2—C8—N2	−177.8 (6)	C19—O4—C18—N4	−175.8 (7)
N1—N2—C8—O1	−1.6 (11)	N3—N4—C18—O3	−0.6 (11)
N1—N2—C8—O2	−179.9 (6)	N3—N4—C18—O4	−179.3 (6)
C8—O2—C9—C10	180.0 (8)	C18—O4—C19—C20	−168.8 (8)

### Hydrogen-bond geometry (Å, °)

**Table:**

*D*—H···*A*	*D*—H	H···*A*	*D*···*A*	*D*—H···*A*
N2—H2A···O3	0.86	2.09	2.913 (7)	161
N4—H4A···O1^i^	0.86	2.09	2.875 (7)	152

Symmetry codes: (i) *x*, −*y*+3/2, *z*−1/2.
